# Limitations of drug registries to evaluate orphan medicinal products for the treatment of lysosomal storage disorders

**DOI:** 10.1186/1750-1172-6-16

**Published:** 2011-04-16

**Authors:** Carla EM Hollak, Johannes MFG Aerts, Ségolène Aymé, Jeremy Manuel

**Affiliations:** 1 Department of Internal Medicine, Division of Endocrinology and Metabolism, Academic Medical Center, Amsterdam, the Netherlands; 2 Department of Medical Biochemistry, Academic Medical Center, Amsterdam, the Netherlands; 3 INSERM SC11, Paris, France; 4 European Gaucher Alliance and Gauchers Association, London, UK

## Abstract

Orphan drugs are often approved under exceptional circumstances, requiring submission of additional data on safety and effectiveness through registries. These registries are mainly focused on one drug only and data is frequently incomplete. Some registries also address phenotypic heterogeneity and natural history data and publications on these aspects have contributed to the knowledge and awareness of these rare diseases. However, for the assessment of long-term outcomes and for cost-effectiveness, the incompleteness and variable quality of the data raises concerns on the usefulness of these registries. The existing registries for orphan drug treatments for lysosomal storage disorders (LSD's) illustrate these limitations. LSD's are inherited disorders of lysosomal metabolism with a wide variety in clinical symptoms, ranging from severe life-threatening neurological disease to mild or even asymptomatic cases. Their prevalence is extremely low and thus data is scarce and scattered all over Europe. In the past few years, several enzyme replacement therapies and an oral substrate inhibitor have been developed which provide lifelong treatment of LSD's. For Fabry disease, two enzymes were authorized at the same time resulting in two different drug registries being required by the European Medicines Agency (EMA) to monitor effectiveness and safety. This has lead to patient data being divided between two separate registries which may have contributed to delays in the assessment of important outcomes. Three treatments (including a recently approved new enzyme) have now been authorized for Gaucher Disease and two other potential therapies are in the pipeline. Dividing up the data on Gaucher disease patients in to five separate registries benefits nobody. We argue that disease specific (rather than drug specific) registries, supervised by independent clinicians are urgently needed for the best long-term evaluation of treatments of these rare diseases.

## Introduction

The European Union enacted the Orphan Drug Regulation in 2000 ( **(EC) No 141/2000 **and **(EC) No 847/2000**) in order to improve the availability of innovative medicines for diseases affecting less than 5/10 000 people. Incentives to pharmaceutical companies include 10 years of market exclusivity, direct access to a Centralized Procedure for Marketing Authorization, scientific advice and fee reductions. Some orphan medicinal products (OMP's), especially those for extremely rare conditions, are approved by the European Medicines Agency (EMA) under "exceptional circumstances" due to the fact that it is unlikely that comprehensive data from clinical trials will become available [1]. Instead, pharmaceutical companies may be required to collect long-term data in a drug registry as part of their authorization. It is important to understand that registries can serve multiple purposes and therefore need different elements. With the increasing use of registries, requirements for each specific purpose are now better defined [2]. For example, registries that serve a public health purpose, such as those that are developed for population surveillance, do not need clinical data beyond diagnosis or follow-up data. On the other hand, registries that are being used to assess the effectiveness and safety of agents after authorization require more stringent elements. For these regulatory drug registries, completeness of case ascertainment, high quality clinical data, verification of data validity and follow-up is mandatory [2]. Existing drug registries for evaluation of the effectiveness of treatments for some orphan diseases have certain limitations in this respect. This has particularly become clear for the lysosomal storage disorders (LSD's). The LSD's comprise a group of very rare disorders which are all due to a deficiency of a lysosomal enzyme and display a variable range of phenotypes. Over the last two decades extremely costly treatments have been developed for these disorders. There are some differences between the LSD's and other orphan diseases for which OMP's were developed that may have contributed to the frequent requirement of drug registries for LSD treatments by the EMA

### Differences between OMP's for lysosomal storage disorders and other indications

In May 2010, the EMA held a conference to evaluate 10 years of Orphan Regulation in Europe. Over the past decade, this legislation has resulted in the approval of 62 OMP's out of > 700 OMP designations [3]. Of these 62 orphan drugs, the majority were for cancer treatments (35.5%), with the next highest being for metabolic diseases and miscellaneous disorders (both 24.2%), followed by cardiovascular and respiratory disorders (8.1%), immunological (4.8%) and musculoskeletal and nervous system disease (3.2%). Post-authorization requirements differed: 54% were authorized without specific requirements, while 41% of OMP's were authorized "under exceptional circumstances" and 5% received "conditional approval", the latter meaning that results of additional studies will be needed for full authorization. Within the relatively large group of metabolic diseases, the majority are LSD's (8 products for 7 diseases, including one product for two diseases and in two cases two enzymes for one disease). These orphan products are mainly enzymes produced in genetically engineered cell lines, which need to be administered intravenously. Oral substrate inhibitors have been developed as well, although miglustat (Zavesca) is the only one that has received approval so far. An authorization "under exceptional circumstances" and a requirement to install post-marketing drug registries are common for OMP's that are indicated for LSD's. With the exception of Myozyme for Pompe's disease, all OMP's for LSD's have been authorized under exceptional circumstances. This is most likely explained by the extreme rarity of LSD's: the prevalence of the diseases for which an OMP has been approved is higher than 1 in 10.000 in more than half of the cases, whereas the prevalence of LSD's vary between 1 in 600.000 and 1 in 37.000 [4-7], see table 1. Since the LSD's for which OMP's are currently available, usually have a slow progressive course and require lifelong treatment, it takes a long time before additional effectiveness and safety data becomes available. The European authorities therefore depend heavily on the outcomes of these registries for final authorization. The need for a post-marketing surveillance system to improve our understanding of long term effectiveness and safety of treatments for LSD's is not debated and LSD registries have proven to add meaningful data [8]. However, the EMA requires separate post-marketing registries for each drug, even when more OMP's are available for one rare disease. In addition, the current drug registries do not sufficiently fulfill the requirements for a regulatory registry. The following examples will illustrate the limitations and challenges of the current drug registries.

**Table 1:** Orphan Medicinal Products for lysosomal storage disorders

**Lysosomal storage disorder (ordered by date of orphan drug designation of 1**^st ^**drug)**	Range of birth prevalence per 100.000 [ref]*	Estimated mean prevalence*	Authorized				Orphan drug designation	
Fabry disease	2.7 [7]	1 in 37.000**	Replagal	Fabrazyme			1-deoxy- galactonojirimycin hydrochloride	

Gaucher disease	1.16-1.75 [4-6]	1 in 70.000	Cerezyme^#^	Zavesca	Vpriv		Taliglucerase alfa	Eliglustat tartrate## Isofagomine tartrate

MPS IH+IS (Hurler/Scheie)	1.14-1.33 [4-6]	1 in 80.000	Aldurazyme					

Glycogen Storage Disease type II (Pompe's disease)	0.17-2.0 [4-6]	1 in 90.000	Myozyme				Rec. adeno-associated viral vector containing human acid alpha-glucosidase-gene	

Mucopolysaccharidosis type VI (Maroteaux-Lamy)	0.15-0.43 [4-6]	1 in 300.000	Naglazyme					

Mucopolysaccharidosis type II (Hunter syndrome)	0.67-1.09 [4-6]	1 in 120.000	Elaprase					

Niemann Pick disease type B	0.10 [6]	1 in 1.000.000					Rec. human acid sphingomyelinase	

Metachromatic Leukodystrophy	1.09-1.85 [4-6]	1 in 70.000					Autologous CD34+ cells transfected with lentiviral vector containing human arylsulfatase A cDNA	Rec.Human Arylsulfatase A

Niemann-Pick Disease, type C	0.35-2.20 [4-6]	1 in 100.000	Zavesca					

Mucopolysaccharidosis, type IIIA (Sanfilippo A syndrome)	0-1.16 [4-6]	1 in 150.000					Recombinant human heparan-N-sulfatase	

Mucopolysaccharidosis, type IVA (Morquio A Syndrome)	0.22-0.6 [4-6]	1 in 250.000					N-terminal hexaglutamine-tagged rec. human N-acetyl galactosamine-6-sulfate sulfatase	Rec. human N-acetylgalactosamine-6-sulfatase

*Birth prevalence values are based upon literature references 4-7, as summarized by Pinto et al [6]. Lowest and highest prevalence values are given (range). The mean prevalence is estimated from these numbers. ** Recent studies point to an increase in birth prevalence of classical Fabry disease [7]. ^#^Cerezyme was licensed before 2000, and is officially not an OMP. ## Eliglustat tartrate has and OD designation as (1R,2R)-octanoic acid[2-(2',3'-dihydro-benzo[1,4] dioxin-6'-yl)-2-hydroxy- 1-pyrrolidin-1-ylmethyl-ethyl]-amide-L-tartaric acid salt. Rec = recombinant

### Fabry disease

Fabry disease is an X-linked lysosomal storage disorder resulting from deficient activity of the enzyme alfa-galactosidase A [9]. Storage of globotriaosylceramide in the vascular wall underlies the pathophysiology [10]. Male patients typically develop painful acroparesthesia followed by proteinuria, renal failure, cardiac hypertrophy and cerebral white matter lesions. Females are carriers and may present a wider range of symptoms. For Fabry disease, two enzymes were granted marketing approval under exceptional circumstances by the EMA in 2002. Both enzymes were tested in small placebo-controlled studies of short duration, that heavily lent on surrogate end points and limited clinical effectiveness parameters [11,12]. Following approval, both pharmaceutical companies, Shire Human Genetic Therapies for Replagal and Genzyme Corporation for Fabrazyme, were asked to provide data for annual reassessment of the benefit/risk profile of their product. Both companies set up a drug registry, (called the Fabry Outcome Survey (FOS) and Fabry Registry respectively). Although each has a board of independent advisors, the pharmaceutical company manages each database. The situation whereby two competitive enzymes were approved at the same time each being evaluated separately resulted in treatment centers being generally involved with only one of the products. In essence, identical datasets were being collected and separate working groups established to address, for example, data on females or children, or effects of treatment on the kidney. It needs to be acknowledged that since these registries did not only address long term effectiveness and safety outcomes, but also focused on natural history data and diversity of disease manifestations, they have added important information. Increased awareness and a better understanding of the natural course of the disease, specific involvement such as the heart, and symptoms in females and pediatric patients, has been achieved [13-18]. The existence of two registries, however, has also resulted in publications that were quite similar [13,14]. Importantly, however, reports from the registries on the longer term outcomes of ERT, (the primary motive behind the requirements for these registries) have been scarce and have only been published using the FOS registry [19,20]. From these publications it is clear that datasets that are complete and accurate enough for analysis are usually extremely small compared to the total number of patients in the registry [20]. This may also be caused by the fact that the quality of the data is variable due to the lack of standardization of assessments. For example, renal function can be measured in different ways with different levels of accuracy [21]. Whether the selection of such a small sample results in a bias is difficult to ascertain, but is certainly a risk. In addition, comparator data from untreated patients is usually lacking. The paucity of high quality long term studies makes it difficult to understand the true health benefit of enzyme therapy in Fabry disease [22]. This is a particular challenge since the disease is highly heterogeneous. For example, it only gradually became clear that in patients with advanced disease the process of deterioration could not be stopped [23-25]. Although it is believed that early therapy could be beneficial, no studies have convincingly shown that occurrence of organ damage can be prevented. How the enzymes compare to each other is another unresolved issue [24]. So far, physicians and researchers have not sufficiently joined forces to initiate multi-center studies independent from industry to address these important longer term outcomes. The lack of collaboration and the separation of data in the two drug registries have undoubtedly contributed to the fact that after almost 10 years experience with Fabrazyme and Replagal, the most important questions such as the benefit of early therapy, the comparison of drugs and the influence of antibodies, remain largely unanswered resulting in having to conduct further clinical studies.

### Gaucher disease

Gaucher disease is the first disorder for which an enzyme therapy was developed. This disorder results from deficient activity of the lysosomal enzyme glucocerebrosidase and is clinically characterized by hepatosplenomegaly, cytopenia with fatigue and bleeding, and devastating bone complications [26]. Cerezyme (imiglucerase) was licensed in Europe in 1994, before the European Orphan Drug legislation was enacted. This treatment has proven to be extremely beneficial in reversing the major manifestations of Gaucher disease [26]. Following this tremendous success, many questions remained unanswered. Since the disorder is extremely heterogeneous, ranging from asymptomatic cases to severe forms in childhood, debates followed on optimal dosing, costs, timing of initiation and prevention of long term complications [27]. The Gaucher Registry was launched in 1991 by Genzyme to collect data on longer term outcomes as well as on the natural history of the disease. Following a Health Technology Assessment conference at the National Institutes of Health (NIH) in 1995, it was suggested that NIH initiate and foster the establishment of a cooperative group of investigators in order to create a patient registry for the analysis of data on natural history and response to therapy [28]. This initiative was not followed through and the Gaucher Registry was further expanded and now contains information on over 5,500 patients. Some interesting publications have arisen from its data, including a number of studies on the variability and rate of improvement of different disease parameters [29-31]. However, variability of assessments and lack of completeness limits its use for robust analyses. For example, in a dose-efficacy analysis, only a subgroup of 366 patients from the registry were analyzed and although the study showed that responses were dose dependent, important outcomes on rates of complication or quality of life could not be extracted [32]. In a study on cost effectiveness of ERT, Connock and coworkers concluded that "Gaucher Registry data, which potentially represented the richest source of observational data for this purpose, were inadequate for the task in hand"[33]. They also expressed concern that the decisions about analyses of Gaucher Registry data largely depend on people who have an interest in the product and recommended that analysis of registry data should be undertaken in such a way that analytical, reporting and publication biases are minimised [33].

Although the Gaucher Registry allows the addition of data on untreated patients as well as on patients treated with competitive products, fragmentation of data with new OMP's entering the market remains a real concern. A second treatment for Gaucher disease with the oral substrate inhibitor Zavesca was licensed in 2002 as an OMP [34]. As part of post-marketing commitments to the EMA, a safety database was set up but this was not designed to evaluate efficacy. Apart from a switch study, no comparative data is available and the registries operate separately. In the near future, data on effectiveness of Gaucher treatment will become increasingly difficult to assess: two new enzymes have recently been developed: velaglucerase (h-GCB, Shire Human genetic Therapies, MA, USA), and taliglucerase (pr-GCD, Protalix Biotherapeutics, Carmiel, Israel). Velaglucerase has recently received marketing authorization in Europe and the EMA has again required, post marketing safety data which will be collected by the company in a drug registry. If this will also be required for taliglucerase and also for the new substrate inhibitor eliglustat (Genzyme Corp) in the course of development, five different drug registries for a single rare disorder will exist. The rapidly increasing number of OMP's for LSD's illustrates that this will only lead to further fragmentation of data (table 1).

### Summary of limitations of drug-registries

#### Quality and completeness of the data

Submission of data to drug registries depends on the willingness and cooperation of individual physicians. As a consequence, the data in these databases may be of variable quality and are usually incomplete. For example, physicians may not enter data or use different criteria to define (say) a bone crisis or radiological abnormality in the skeleton in Gaucher disease. Biomarker analyses are usually impossible to analyze as the variability in assays produce insurmountable hurdles. In addition, the datasets are usually narrowly focused and some important outcome data may be missed.

#### Lack of transparency

Firstly, as mentioned, the pharmaceutical companies manage the registries, with boards consisting of treating physicians and scientists having a role in formulating research questions. However, the analyses are performed by statisticians and epidemiologists employed or commissioned by the pharmaceutical company. The board will discuss the results of analyses, but will usually not have access to the source data from the registry. This problem could be overcome by using an independent group of statisticians and/or epidemiologists or by granting free access to the data on request. This requires rules that are agreed upon by the clinicians, the patient organizations, the regulatory agencies and the pharmaceutical company.

Second, the yearly reports that are submitted to EMA are not open to the public. The entire discussion on safety and effectiveness is between the company and the EMA. Only when safety issues require the issuing of a "Direct Healthcare Professional Communication", will the community be informed. The recent shortage of imiglucerase and agalsidase beta and subsequent issues with enzyme supplies were only disclosed at a late stage. This was partially to do with the obligations of the company to inform and receive guidance from EMA first and led to EMA issuing guidelines to the Gaucher and Fabry community that were not discussed with the treating physicians and were also not in line with expert opinion [35]. Data on the effect of the shortage are difficult to extract from the drug registries and understanding the clinical effects of switching to alternative treatments is an even bigger challenge.

#### Fragmentation of data

When more than one product for an orphan disease reaches the market, post-marketing requirements to set up a drug registry will inevitably result in fragmentation of data and lack of collaboration between treatment centers. In addition, studies on cost-effectiveness are carried out on a national level instead of a European level, usually initiated as a result of concern over the high price of the drugs. A huge amount of governmental money is spent on analyses of national datasets that are too limited to give meaningful results. If outcomes result in abandoning access to some of the drugs from the health care reimbursement system, this will be on the basis of insufficient data.

## Conclusions

We conclude that collaboration on a European level and between all stakeholders is needed to improve the evaluation of orphan drugs for LSD's. To avoid fragmentation of data, disease registries, rather than drug registries should be established. Such registries should facilitate not only the collection of high quality data on safety and effectiveness for each drug [2], but should also compare outcomes. More importantly, with a board of independent experts in the lead, consisting of physicians, patient representatives, epidemiologists and statisticians, uniformity of evaluation should be guaranteed. A major challenge is to enhance the quality and completeness of the data, which needs systems and resources for data validation and management. To achieve this, systems should be developed that support physicians to submit data, in line with defined requirements [3]. When physicians are not reporting to the registry, they might consider transferring their patients to a center of expertise that does. These registries should be supervised and analyses performed by an independent body and access to data should be possible on request. They should be audited at regular intervals. This approach will save money for both industry and national governments, as instead of supporting multiple registries, there would be only one. As there is a mutual interest from companies, physicians and governments the financial burden should be shared through a public-private partnership, in the best interest of the patients and of the community at large.

## List of abbreviations

h-GCD: Gene-Activated Human Glucocerebrosidase; pr-GCD: Plant Cell Expressed Recombinant Human Glucocerebrosidase.

## Competing interest statement

CEMH participates in the Fabry Outcome Survey (FOS, sponsored by Shire HGT) and the Gaucher Registry (sponsored by Genzyme Corp) but her views are not influenced by these relationships. She has received reimbursement of expenses and honoraria for lectures on the management of lysosomal storage diseases from Genzyme Corporation, Shire and Actelion. All honoraria are donated to the Gaucher Stichting, a national foundation that supports research in the field of lysosomal storage disorders. JMFGA has received travel reimbursements from Genzyme, Actelion and Shire for invited lectures at scientific meetings. He has no conflict of interest to declare. SA is a medical geneticist, director of research at INSERM and head of Orphanet, the International information portal on rare diseases and orphan drugs. Orphanet as an institution receives occasionally grants from Industry. Currently "LFB Biomédicaments" sponsors the production and update of emergency guidelines and texts for the French encyclopaedia for general public. "Shire" sponsors the development of the Orphanet encyclopaedia. "Alexion" sponsors the translation of the emergency guidelines from French.into other language. JM is a practicing English lawyer and is Chairman of the European Gaucher Alliance and the UK Gauchers Association. The European Gaucher Alliance receives funding from the pharmaceutical Industry and has a policy of not requesting more than 30% of its budget from any one pharmaceutical company. The UK Gaucher Association does not accept funding from industry but companies are occasionally invited to sponsor individual projects or fundraising activities. JM has received reimbursement for travel and expenses for attendance at meetings. All honoraria received are donated to either the EGA or the UK Association.

## Authors' contributions

CEMH has conceived, designed and drafted the manuscript. JMFGA, SA and JM have been involved in revising it critically for important intellectual content. All have given final approval of the version to be published
